# A Thermostable Oral SARS-CoV-2 Vaccine Induces Mucosal and Protective Immunity

**DOI:** 10.3389/fimmu.2022.837443

**Published:** 2022-02-25

**Authors:** Bertrand Bellier, Alicia Saura, Lucas A. Luján, Cecilia R. Molina, Hugo D. Luján, David Klatzmann

**Affiliations:** ^1^ Sorbonne Université, INSERM, UMRS 959, Immunology-Immunopathology-Immunotherapy, i3, Paris, France; ^2^ Centro de Investigación y Desarrollo en Inmunología y Enfermedades Infecciosas (CIDIE), Consejo Nacional de Investigaciones Científicas y Técnicas (CONICET)/Universidad Católica de Córdoba (UCC), Córdoba, Argentina; ^3^ Facultad de Ciencias de la Salud, Universidad Católica de Córdoba (UCC), Córdoba, Argentina; ^4^ AP-HP, Hôpital Pitié-Salpêtrière, Clinical Investigation Center for Biotherapies (CIC-BTi) and Immunology-Inflammation-Infectiology and Dermatology Department (3iD), Paris, France

**Keywords:** COVID-19, vaccine, VLP (virus-like particle), oral vaccination, mucosal immunity

## Abstract

An ideal protective vaccine against SARS-CoV-2 should not only be effective in preventing disease, but also in preventing virus transmission. It should also be well accepted by the population and have a simple logistic chain. To fulfill these criteria, we developed a thermostable, orally administered vaccine that can induce a robust mucosal neutralizing immune response. We used our platform based on retrovirus-derived enveloped virus-like particles (eVLPs) harnessed with variable surface proteins (VSPs) from the intestinal parasite *Giardia lamblia*, affording them resistance to degradation and the triggering of robust mucosal cellular and antibody immune responses after oral administration. We made eVLPs expressing various forms of the SARS-CoV-2 Spike protein (S), with or without membrane protein (M) expression. We found that prime-boost administration of VSP-decorated eVLPs expressing a pre-fusion stabilized form of S and M triggers robust mucosal responses against SARS-CoV-2 in mice and hamsters, which translate into complete protection from a viral challenge. Moreover, they dramatically boosted the IgA mucosal response of intramuscularly injected vaccines. We conclude that our thermostable orally administered eVLP vaccine could be a valuable addition to the current arsenal against SARS-CoV-2, in a stand-alone prime-boost vaccination strategy or as a boost for existing vaccines.

## Introduction

Current field observations show that a protective vaccine against SARS-CoV-2 is likely the only means of controlling the pandemic (1–3). To fulfill this promise, these vaccines should ideally be effective in preventing infection and virus transmission and, importantly, well accepted by the population. In underdeveloped countries, vaccines should also have a simple logistic chain (4, 5).

Regarding efficacy, years of vaccine research have demonstrated that vaccine protective effects rely in large part on systemic neutralizing antibodies, while local cytotoxic T cell responses are for the most part responsible for virus eradication after a productive infection (6). Moreover, for upper respiratory tract infection, a robust mucosal immunity is likely required to minimize virus transmission (7–9). Regarding vaccine hesitancy, oral administration would favor acceptance and minimize the risk of adverse events (10). Regarding logistics, oral administration would also ease mass vaccination, and a thermostable vaccine would ensure a much-simplified logistic chain. Likewise, an optimal vaccine against SARS-CoV-2 should be thermostable, orally administered and able to induce a robust mucosal neutralizing immune response.

To tackle the challenge of producing such a vaccine, we used our platform based on retrovirus-derived enveloped virus-like particles (eVLPs) that has been developed to generate neutralizing antibody (NAb) (11). Indeed, these eVLPs have the same lipid membrane as the cell they derive from. Likewise, virus envelope proteins that eVLPs express have the same conformation as they have on the lipid membrane of an infected cell, and on the virus itself. As NAbs are mostly targeted to conformational structures, eVLPs are thus particularly suitable for NAb induction (12). We previously showed that such eVLPs could generate robust NAbs against many viruses, such as influenza, HCV and CMV, in mice, macaques and humans (13–17). Moreover, exploiting the versatile engineering possibilities for these eVLPs, we recently showed that they could be harnessed with variable surface proteins (VSPs) from the intestinal parasite *Giardia lamblia*, affording them resistance to degradation and the triggering of robust mucosal cellular and antibody immune responses after oral administration (18, 19). Notably, as previously described, VSP has also an intrinsic adjuvant effect (18, 19).

We used this experience to design and evaluate a thermostable orally administered eVLP vaccine against SARS-CoV-2. We tested the expression of various forms of the Spike protein (S) with or without SARS-CoV-2 membrane protein (M) expression. We found that eVLPs expressing a pre-fusion stabilized form of S plus M trigger robust mucosal NAbs against SARS-CoV-2 in mice and hamsters, which translate into complete protection from a viral challenge. We consider that such a vaccine could be part of the arsenal against SARS-CoV-2, in a stand-alone prime-boost vaccination strategy or as a boost for existing vaccines.

## Materials and Methods

### Viruses

SARS-CoV-2 isolates were propagated in Vero E6 cells in Opti-MEM I (Invitrogen, Cat. # 51985091) containing 0.3% bovine serum albumin (BSA) and 1 μg of L-1-tosylamide-2-phenylethyl chloromethyl ketone-treated trypsin per mL at 37°C.

### Experimental Animals

For immunization and challenge, the group sizes were chosen based on previous experience and littermates of the same sex were randomly assigned. The number of animals for each experiment and all procedures followed the protocols approved by the Institutional Committee for Care and Use of Experimental Animals. Six week- or four month-old male and female BALB/c mice were used for initial experiments, 6-month-old female and male Golden Syrian hamsters were used in the immunization studies, and 1-month-old female and male SPF Golden Syrian hamsters were used in the challenge experiments. For challenge experiments, under ketamine−xylazine anesthesia, ten hamsters per group were inoculated with 10 (5) PFU of SARS-CoV-2 (in 100 μL) or PBS (mock) *via* the intranasal route (20, 21). At 24h post-inoculation, each challenged animal was transferred to a new cage and co-housed with one naïve hamster to assess their contagiousness. Nasal washes were collected in naïve animals on days 5 and 10 post-contact for virus detection. In challenged animals, no blinding and washes were done and body weights were measured were monitored for 28 days (21).

### eVLP Expression Plasmids

For pGag, the cDNA sequence encoding the MLV Gag (Uniprot: P0DOG8.1) without the C-terminal Pol sequence was used (19). For SARS-CoV-2 spike protein variants, the cDNA sequences were cloned in the phCMV expression vector (19). All plasmids were verified by sequencing as reported. The SARS-CoV-2 spike protein variants derived from the wild type strain Swt (NC_045512, original Wuhan variant) all having the D615G and the 682RRAR-685GSAS (modFurinCS) mutations. Additional mutations were inserted in specific variant: Sst1: K986P/V987P; Sst2: T791C/A879C; Sst3: S884C/A893C; Sst4: G885C/Q913C; Sst5: S884C/Q913C.

### eVLP Generation, Production, Purification, and Validation

*e*VLPs were produced by transient transfection of either HEK293 cells or HEK293-1267 cells, with pGag, pS or its variants, and pM plasmid DNA, using PEI as transfection reagent. Cells were transfected at 70% confluence in T175 flasks with 70 μg of total DNA per flask at a PEI: DNA mass ratio of 3:1. VLP-containing supernatants were harvested 72 h post-transfection, filtered through a 0.45 μm-pore size membrane, and concentrated 20 x in a centrifugal filter device (Centricon^®^ Plus-70-100 K, Millipore Cat. # UFC710008) and purified by ultracentrifugation through a 20% sucrose cushion in an SW41T Beckman rotor (25,000 rpm, 4 h, at 4°C). Pellets were resuspended in sterile TNE buffer (50 mM Tris-HCl pH 7.4, 100 mM NaCl, 0.1 mM EDTA). Proteins were measured using the Bradford method. The samples were analyzed using a Nanosight NS300 in a light scatter mode. The nanoparticle tracking analysis software (NTA 3.1) defined the concentration, size, and intensity of the particles within the samples. Direct immunofluorescence assay using 7F5-FITC was performed to confirm the presence of VSP onto eVLPs. Specific ELISA assays were performed to quantify the spike proteins on the surface of the different eVLPs. For western blotting, proteins were resolved by 10% SDS–PAGE and transferred onto PVDF membranes before incubation with specific primary antibodies. Alkaline phosphatase-conjugated secondary antibodies were used and were detected by BCIP/NBT substrate (19).

### Immunizations

Mice and Golden Syrian Hamsters were fasted for 4 h and then orally immunized with two weekly doses of 100 μg of different eVLPs. For IM immunization, two weekly doses of 10 μg of different eVLPs were administered. Animals from the negative control group (naive) received oral immunizations with vehicle alone.

### Fluid Collection

Blood was collected weekly from the retro-orbital sinus of hamsters and serum was separated and stored at −80°C. No animals were harmed during the collection of blood. BAL was collected through the trachea by injection-aspiration of 1 mL of PBS with protease inhibitors.

### Neutralization Assays

Serum taken from immunized hamsters, 2 weeks post-immunization, was tested for viral neutralizing antibody titer by microneutralizing assay in Vero E6 cells. Briefly, dilutions of serum samples (1:50 to 1:10,000) were mixed with 100 TCID50 of SARS-CoV-2 virus and incubated at 37°C for 1 h. The mixture was then added to Vero E6 cells and further incubated at 37°C for 72 h (22). The neutralizing antibody titer was defined as the highest dilution that inhibits 50% of the cytopathic effect.

### Enzyme-Linked Immunosorbent Assay (ELISA) Tests

The levels of IgG and IgA antibodies against spike protein were determined by ELISA by sensitizing the plate with homogenates of killed whole virus produced *in vitro*. Spike was quantified using purified protein (Human coronavirus HCoV-229E Spike Protein (S1+S2 ECD). Sino Biological, Inc. 40605-V08B and Spike Protein (Active Trimer) R&D Cat. # 10549-CV). The following secondary antibodies were used: Mouse anti-Hamster IgG Cocktail, Clone: G94-56, G70-204 (BD Biosciences, Cat. # 554009); Mouse anti-Hamster IgM, Clone: G188-9, (BD Biosciences Cat. # 554035); Hamster Immunoglobulin A (IgA) ELISA Kit (MyBiosources Cat. # MBS029668); Mouse monoclonal (H6) anti-SARS-CoV-2 spike glycoprotein (Abcam Cat. # ab273169).

### Statistical Analyses

Prism (GraphPad Software) was used to perform unpaired Mann-Whitney test on datasets. Each data set corresponds to independent experiments. All figures show mean ± S.E.M. Statistically significant differences are indicated in each graph as ^*^ *p*< 0.05, ^**^ *p*< 0.01 and ^***^ *p*< 0.001 and ns = not significant.

## Results

### Designing and Selecting the Immunogens

**Figure 1:**
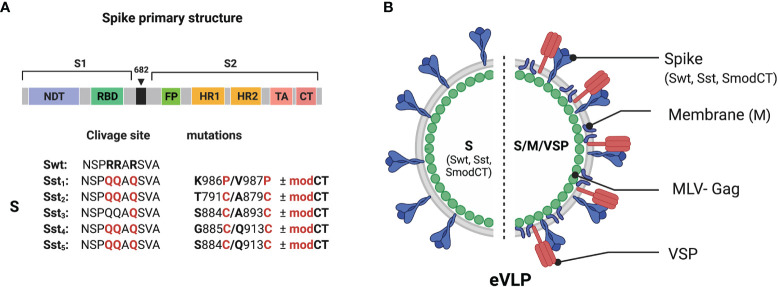
Structure and organization of the SARS-CoV-2 vaccine. **(A)** Linear diagram of the sequence and structure elements of the SARS-CoV-2 spike proteins used as immunogens. Structural elements include the S1 and S2 ectodomains derived from the original Wuhan variant (Swt) in which specific mutation were inserted. The native furin cleavage site was mutated (RRAR ➔ QQAQ) in all variants (Sst_1_-Sst_5_) to be protease resistant. Specific substitution (in red) and respective position were indicated. The spike variants with a CT modified to abolish ER retention (modCT) were also generated. **(B)** SARS-CoV-2 eVLP structure. Native or stabilized form of the SARS-CoV-2 spike (Swt or Sst) were pseudotyped on eVLP formed with the viral matrix protein MLV-Gag in association or not with the SARS-CoV-2 M proteins and the VSP from the intestinal parasite Giardia lamblia.

**Figure 2:**
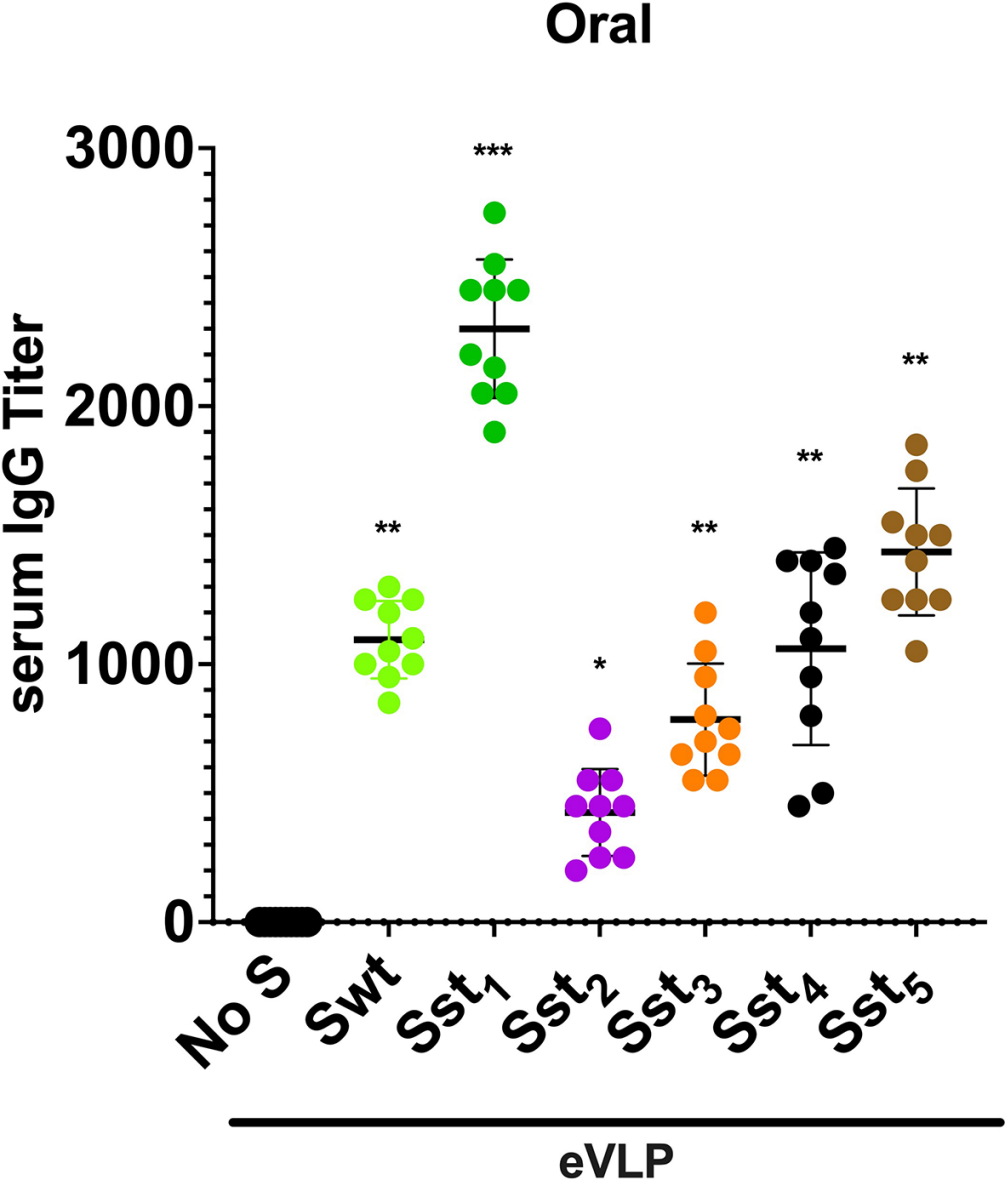
Immunogenicity of different variants of SARS-CoV-2 spike-eVLPs in mice after oral immunization. VSP-pseudotyped eVLPs displaying the following SARS-CoV-2 spike protein variants (Sst1, Sst2, Sst3, Sst4, Sst5) or wild-type sequence (Swt) were produced and used for oral immunization in mice as described in Methods. No S means eVLPs without spike. Values represent the IgG titer in blood of each animal and the horizontal line indicates the mean value. Stabilized Spike 1 (Sst1) displayed onto eVLPs elicited the higher titers and was selected for subsequent experiments. *p < 0.05, **p < 0.01 and ***p < 0.001 in comparison to control group (No S, n = 10).

Initially, the spike protein S of SARS-CoV-2 was evaluated and several variants for stabilization of the receptor-binding domain (RBD) and stabilization in the pre-fusion state were designed (**Figure 1A**). Point mutations, Cys-molecular clamps, furin-cleavage site elimination and Proline (Pro) substitutions (23, 24) were generated and cloned. Spike protein variants that conserved their own cytoplasmic tail (CT), although it can be advantageous to swap it for the CT of VSV-G to improve pseudotyping onto eVLPs, or that were modified to delete its ER retention signal were also designed. Then, those eVLPs were produced and validated for the correct composition as described (19). VSP-pseudotyped eVLPs were orally administered to BALB/c mice and the level of serum IgGs was determined by ELISA (**Figure 2**).

Notably, spike variants lacking the furin-cleaved site and with the two Pro substitutions (Sst1), as reported by Wrapp et al. (25), including the D614G mutation (26, 27), was the most efficient in eliciting a high level of antibodies. Identical experiments were performed in ob/ob mice (JAX™ Mice Strain), db/db mice (JAX™ Mice Strain) and aged mice (>4 month-old) of both sexes and yielded similar results (not shown), indicating that antibody production after oral immunization with eVLPs does not vary according to underlying condition, sex or age of the mice. Therefore, Sst1, the most effective variant of S, was selected for further studies.

Given that glycosylation of spike would be important for its appropriate conformation since there are numerous glycosylated sites near the RDB (28, 29), glycosylation of S should likely influence the generation of efficient NAbs. As the C-terminal CT of the SARS-CoV-2 spike is important in proper glycosylation, spike with a CT modified to abolish ER retention (SmodCT) was generated. In addition, the envelope membrane protein M is known to retain S at the ER for improvement of the first steps of glycosylation and, subsequently, remains attached to the CT of S during their journey throughout the Golgi apparatus, where final glycosylation is accomplished (30). Consequently, eVLPs with protein M of SARS-CoV-2 were also tested (**Figure 1B**). Although that was the main reason for including M in the eVLPs, subsequent reports showed a specific T cell response to several epitopes of this protein in patients who recovered from COVID-19 (31–33). Thus, incorporation of M in the envelope of the eVLPs could not only benefit proper glycosylation of S, but also the production of a stronger cellular response to the virus. Therefore, eVLPs with or without M and with and without a modified CT were generated. Finally, all these eVLPs were generated with or without the incorporation onto the eVLP surface of a VSP derived from *Giardia* (18, 19).

### eVLP Immunogenicity After Intramuscular Injections

**Figure 3:**
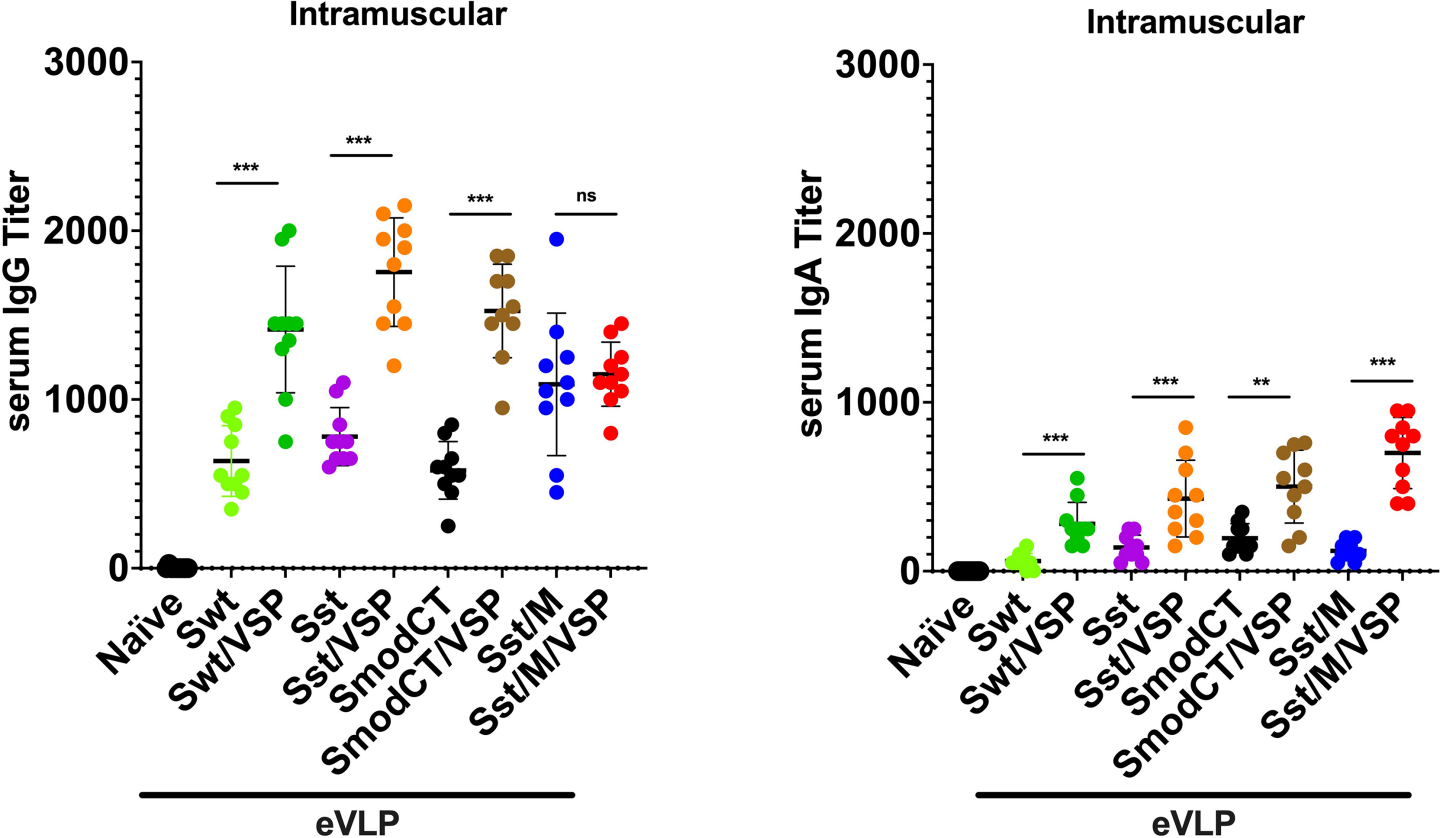
Serum antibody responses to intramuscular administration of different vaccine formulations in hamsters. Serum IgG (left) and serum IgA (right) titers of hamsters unvaccinated (naïve) or vaccinated intramuscularly with different formulations. Wild-type spike (Swt), stabilized S as in **Figure 1** (Sst) and the same in which the cytoplasmic tail was modified (SmodCT) were used to pseudotype eVLPs including or not SARS-CoV-2 M proteins and *Giardia* VSPs. Values represent the mean ± s.e.m. **p < 0.01; ***p < 0.001; ns, not significant; Mann Whitney test comparing hamsters immunized with eVLPs pseudotyped or not with *Giardia* VSPs (n=10).

eVLPs administered intramuscularly (i.m.) to hamsters, whether or not decorated with VSPs, induced high levels of IgG and moderate level of IgA in serum (**Figure 3**), validating these immunogens. Noteworthy, the presence of the *Giardia* VSP on the eVLPs promoted higher levels of antibodies than the plain eVLP (p<0.01 or 0.001; except for IgG with i.m. VLPs Sst/M), highlighting the adjuvant effect of the VSPs (18, 19).

The levels of serum IgG after immunization with Sst1 were higher than with the wild-type spike [eVLP Swt/VSP - eVLP Sst/VSP; p<0.05, Mann Whitney; **Figure 3**], and the addition of M further induced a slight but not significant increase of the IgG response [eVLP Sst - eVLP Sst/M; p=0.07, Mann Whitney; **Figure 3**]. The levels of serum IgGs were also higher than those of IgA were, as expected. However, these values are in the same range those obtained after immunization with other vaccine formulations (34, 35) or those found in plasma from convalescent patients (36, 37).

Altogether, these results clearly show that plain eVLPs are already good immunogens when administered by injection in the absence of any adjuvant, highlighting that eVLPs are structures well recognized by the immune system. These responses are strongly increased when VSPs were present on eVLPs according to the VSPs’ intrinsic TLR4-dependent adjuvant properties (18, 19).

### eVLP Immunogenicity After Oral Administration

**Figure 4:**
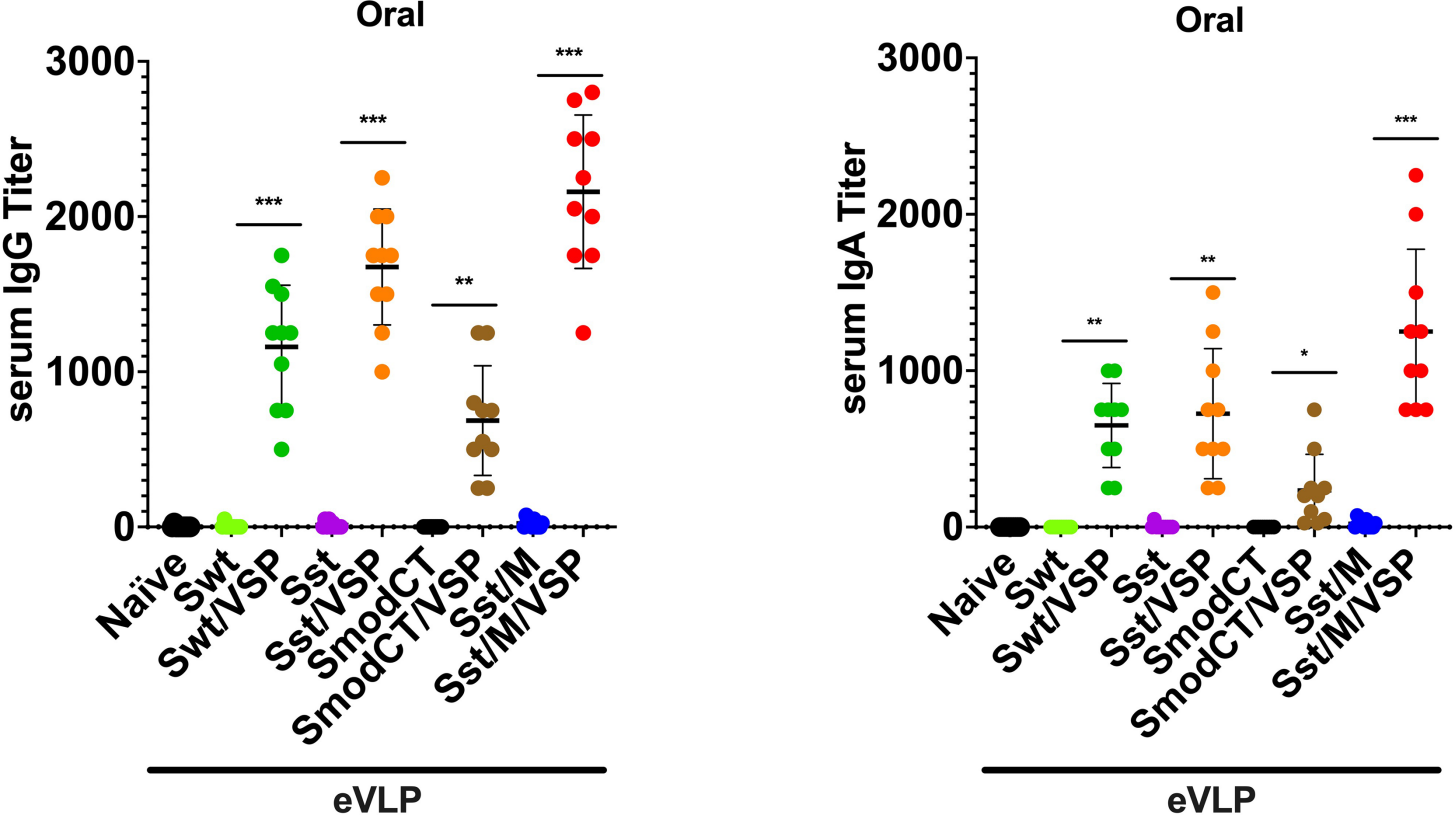
Serum antibody responses to oral administration of different vaccine formulations in hamsters. Serum IgG (left) and serum IgA (right) titers of hamsters unvaccinated (naïve) or vaccinated orally with different formulations. Wild-type spike (Swt), stabilized S as in **Figure 1** (Sst) and the same in which the cytoplasmic tail was modified (SmodCT) were used to pseudotype eVLPs including or not SARS-CoV-2 M proteins and *Giardia* VSPs. Values represent the mean ± s.e.m. *p < 0.05; **p < 0.01; ***p < 0.001; Mann Whitney test comparing hamsters immunized with eVLPs pseudotyped or not with *Giardia* VSPs (n=10).

Oral administration of the same validated immunogens showed that the absence of the *Giardia* VSP decorating the different eVLPs led to no detectable immune response (**Figure 4**), most likely due to destruction of the eVLPs in the upper small intestine. Additionally, the modification of the CT of S appeared detrimental in inducing either serum IgG (**Figure 4** left) or IgA (**Figure 4** right) as compared with S having the wild-type CT. However, serum IgG and IgA titers were augmented when M was incorporated into the VSP-eVLPs (**Figure 4**). Noteworthy, the serum IgA induced by the VSP-eVLPs were higher after oral administration (**Figure 4** right) than after i.m. injections (**Figure 3** right).

Altogether, these results show that the VSPs are essential for oral immunization with eVLPs and the immunogenicity of orally administered eVLPs was strictly dependent on the presence of the VSP on their surface, highlighting the efficiency of this route of immunization, which provides slightly higher Ig responses than after i.m. injection of the same immunogens.

### eVLP-Induced Mucosal Immunity

**Figure 5:**
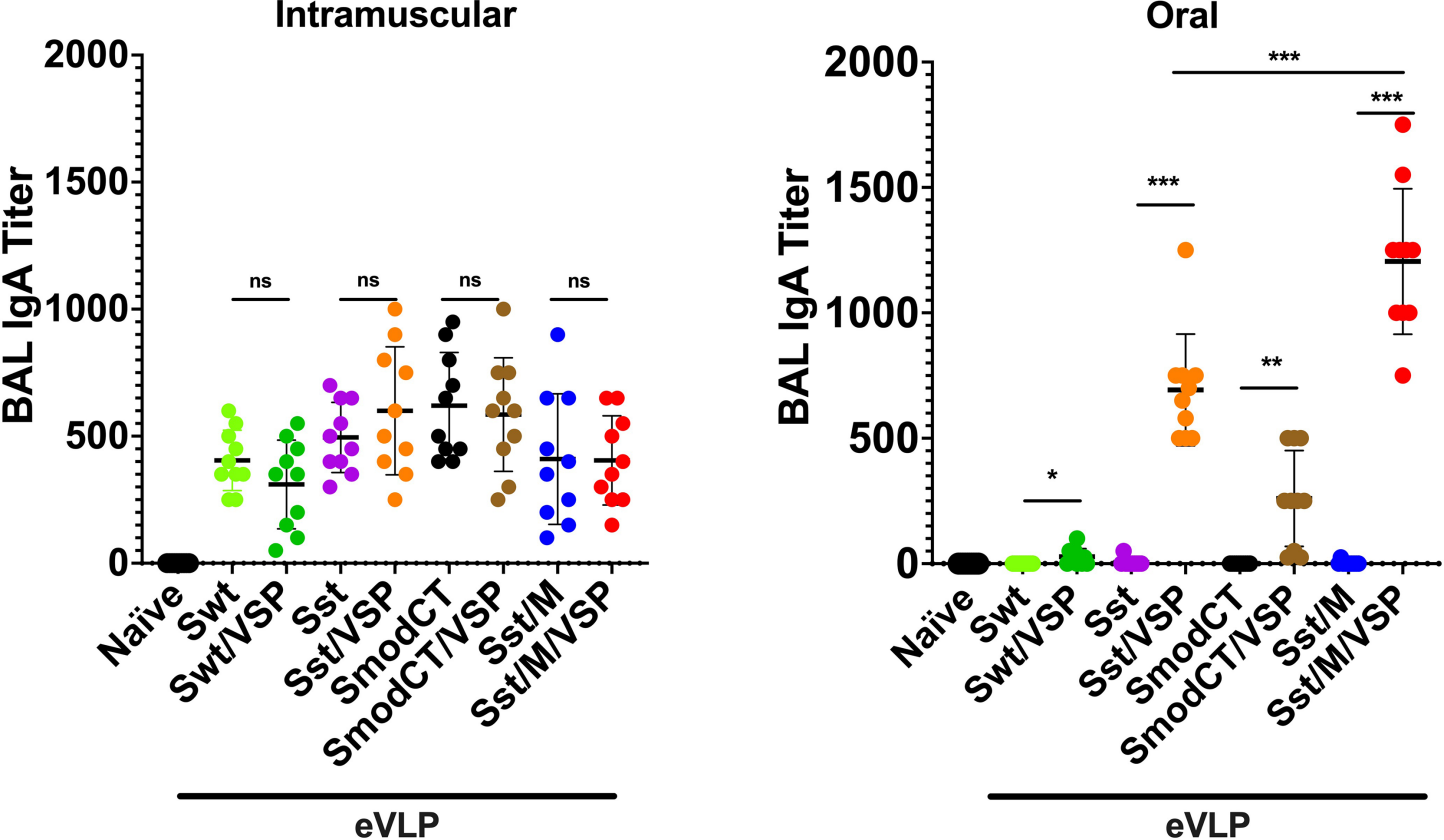
Bronchoalveolar lavage IgA responses in hamsters vaccinated intramuscularly or orally. Bronchoalveolar lavage IgA titers of hamsters unvaccinated (naïve) or vaccinated intramuscularly (left) or orally (right) with different formulations. Wild-type spike (Swt), stabilized S as in **Figure 1** (Sst) and the same in which the cytoplasmic tail was modified (SmodCT) were used to pseudotype eVLPs including or not SARS-CoV-2 M proteins and *Giardia* VSPs. Values represent the mean ± s.e.m. *p < 0.05; **p < 0.01; ***p < 0.001; ns, not significant; Mann Whitney test comparing hamsters immunized with eVLPs pseudotyped or not with *Giardia* VSPs (n=7 to 10).

When the presence of IgA was analyzed in bronchoalveolar lavages (BAL) of animals immunized orally or by i.m. injection, it was noticed that intramuscularly immunized animals have a consistently low level of IgA titers. In contrast, those administered orally showed high titers of IgA but only with eVLPs containing VSP, M and stabilized S (**Figure 5**). Again, these results confirm (i) the high immunogenicity of eVLP formulations, (ii) the crucial role of VSPs in protection of eVLPs, and (iii) the higher efficiency of oral administration in inducing mucosal IgA.

### eVLP-Induced Neutralizing Antibodies

**Figure 6:**
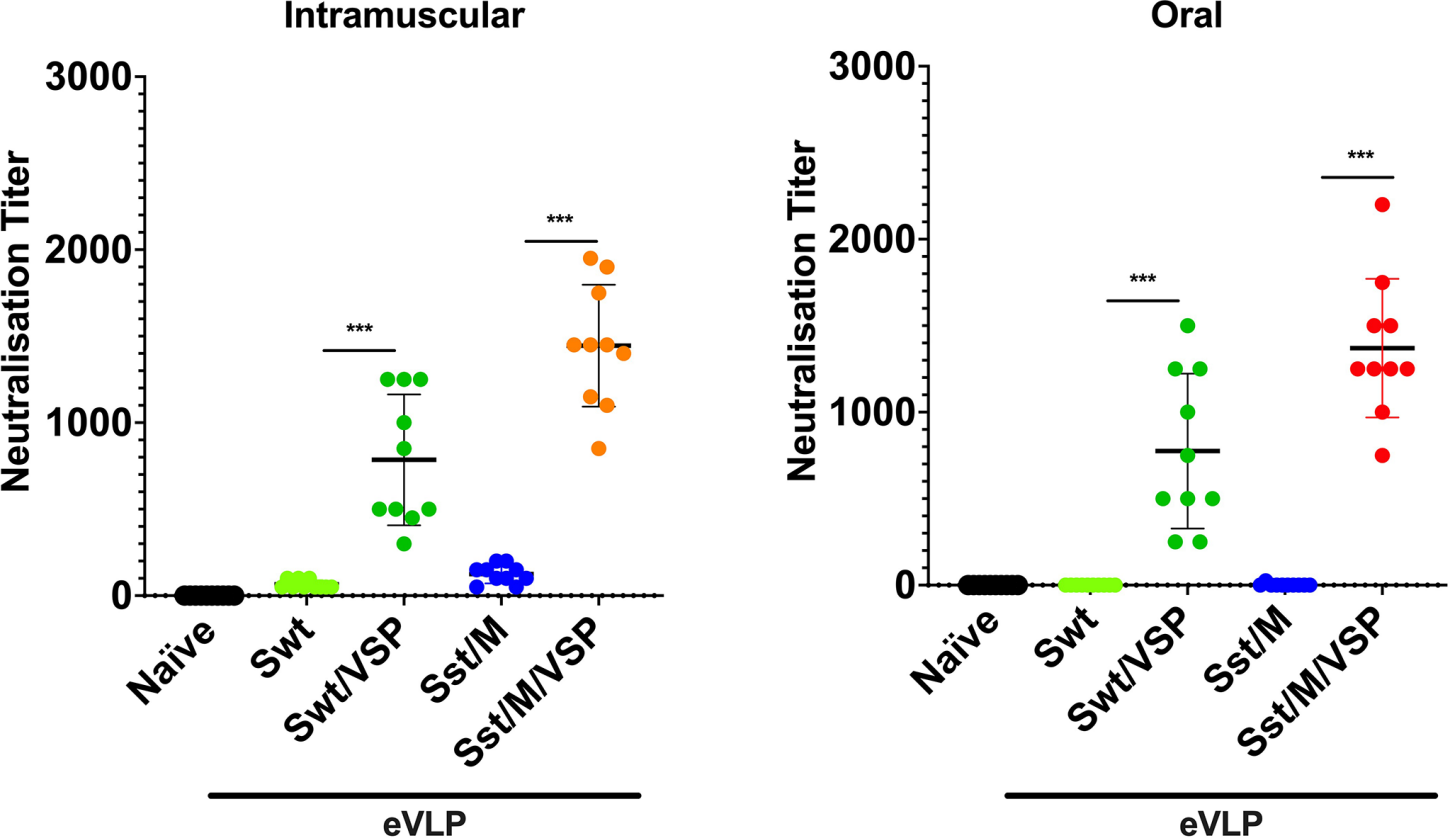
Neutralizing antibodies against SARS-CoV-2 entry induced in hamsters vaccinated intramuscularly or orally. Neutralizing antibody titers of intramuscularly (left) and orally (right) vaccinated animals with selected eVLP formulations and control animals (naïve). Values represent the mean ± s.e.m. ***p < 0.001. Mann Whitney test comparing hamsters immunized with eVLPs pseudotyped or not with *Giardia* VSPs (n = 10).

We then compared the best eVLPs expressing the Sst1 spike and M proteins (eVLP Sst/M/VSP) with the eVLPs expressing a wild-type spike (eVLP Swt/±VSP) for the generation of neutralizing antibodies. Interestingly, there was no NAb generated after i.m. injection when the VSP was not present on the eVLPs (**Figure 6**), highlighting its adjuvant effect. With the VSPs present, both wild-type and stabilized S was able to generate NAbs, as observed for the different commercial vaccines already being administered to humans (1, 38–40). Remarkably, the titer of NAbs generated after oral administration are equivalent to those generated after i.m. injections (**Figure 6**), highlighting the efficiency of VSP-eVLPs as immunogens.

### eVLP as Booster Immunization

**Figure 7:**
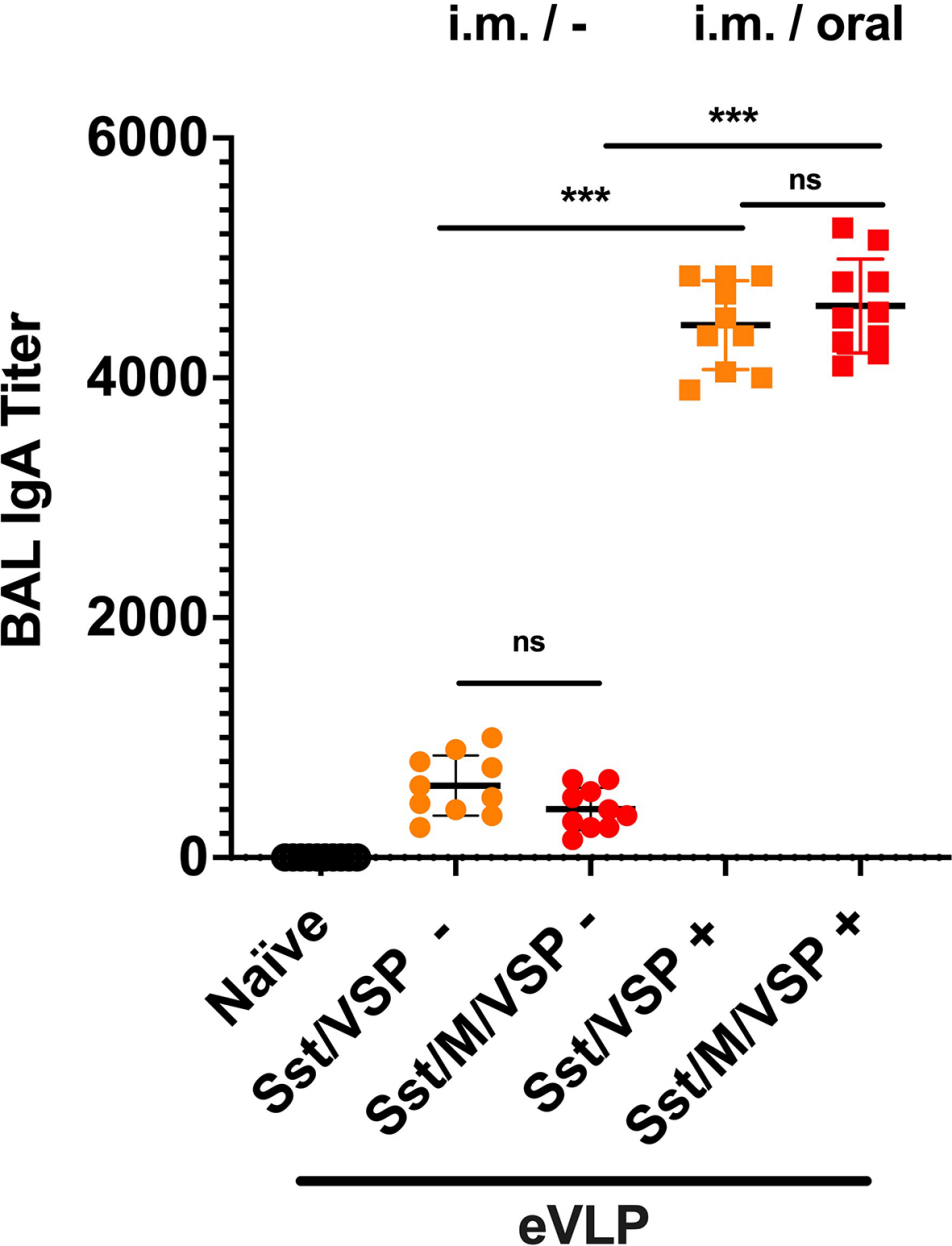
Bronchoalveolar IgA after an oral boost of intramuscularly vaccinated hamsters. Animals from **Figure 5A**, immunized by i.m. injection of VLPs pseudotyped with stabilized spike and VSP and formed in the presence or absence of SARS-CoV-2 M protein, were then boosted (+, squares) or not (-, cercles) with the same VLPs by oral route. Values represent the mean ± S.E.M. ***p < 0.001; ns, not significant; Mann Whitney tests comparing hamsters immunized with eVLPs formed with M or not and comparing hamsters orally boosted or not (n= 10).

Given the incomplete level of protection afforded by some vaccines and the constant emergence of new viral variants, these orally administered immunogens could possibly be good boosts for existing vaccines. For these reasons, an oral boost was applied to animals previously vaccinated by i.m. injections of eVLPs (eVLP Sst/ ± M/VSP). In these animals, a third dose of the oral formulation with the same eVLPs containing VSP, stabilized S ± M induced a major increase in the levels of IgA in BAL as compared to those that only received two doses intramuscularly (**Figure 7**). No statistical difference was observed between eVLPs that included M or not.

### Oral Vaccination With VSP-eVLPs Protects Hamsters From a Challenge With SARS-CoV-2

**Figure 8:**
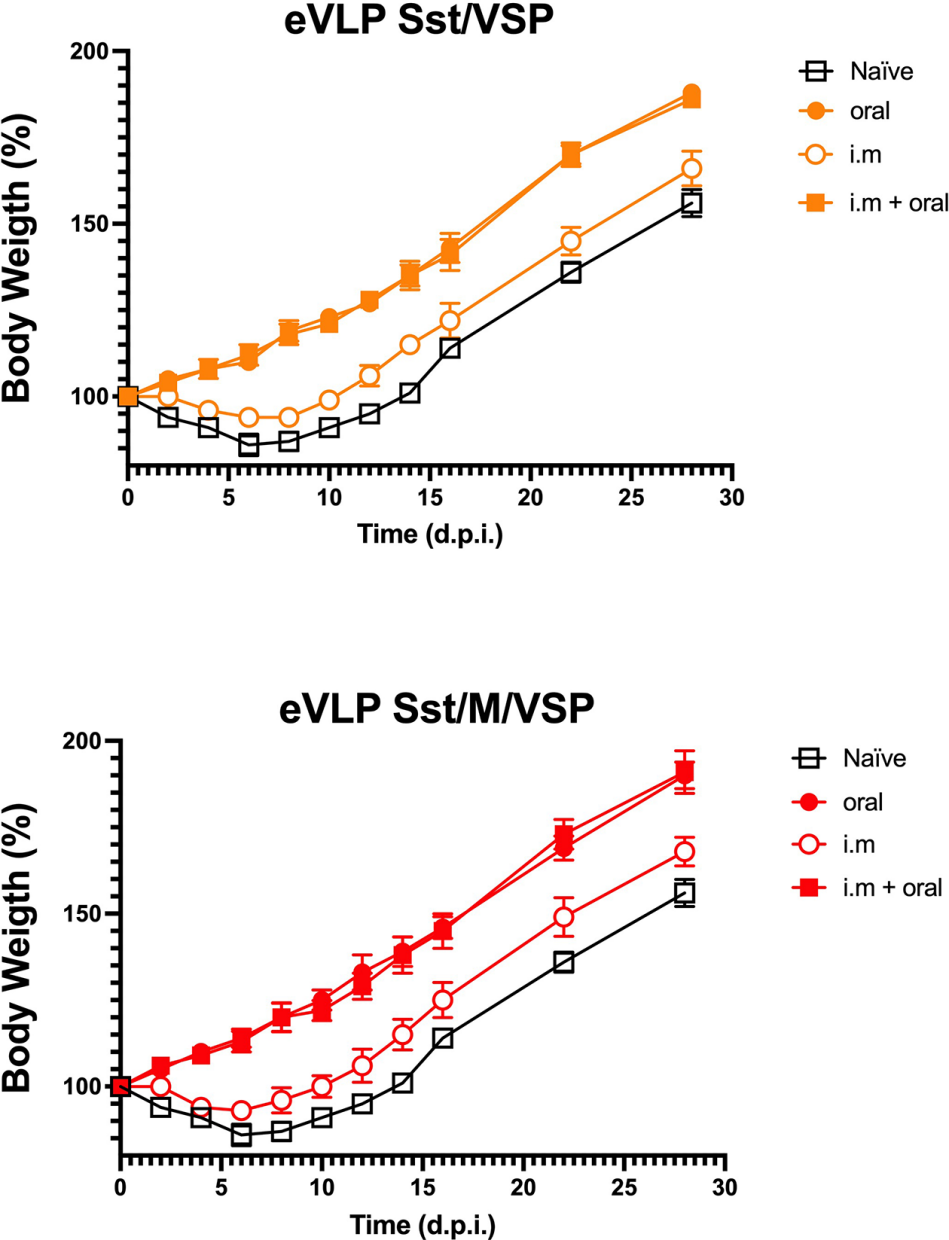
Vaccinated hamsters challenged with SARS-CoV-2. Hamsters either orally or intramuscularly vaccinated with eVLPs expressing VSP and stabilized S with the addition or not of the M protein (eVLPs Sst/VSP, up; eVLPS Sst/M/VSP, down) were challenged intranasally with purified SARS-CoV-2 virus. Every two days, the weight and general status of the unvaccinated animals (open squares), the orally (closed cercle) or intramuscularly vaccinated (open cercle) were monitored and recorded. Additionally, intramuscularly vaccinated animals orally boosted with the same formulations were included and monitored (closed squares). Values represent the mean of three independent determinations made 1 h apart ± SD for each animal (n= 10/group).

Animals immunized with eVLPs in which stabilized S and VSP were present on the particles (eVLP Sst/ ± M/VSP) were challenged with SARS-CoV-2 and the clinical response of the hamsters was determined by monitoring their weight (21). Control animals lost weight during the two weeks following the viral challenge (**Figure 8**) and then recovered, as reported for experimental infections in hamsters (20, 21). Hamsters that were only immunized intramuscularly were not fully protected as they had only a slightly lower weight loss. In contrast, oral immunization with VSP-eVLPs fully prevented weight loss, whether or not the M protein was present, and similarly to animals that were immunized by injection first and then boosted orally (**Figure 8**). Interestingly, orally vaccinated animals were not contagious, unlike intramuscularly vaccinated animals. Indeed, they did not transmit the virus to naïve hamsters housed in the same cage, as shown by the absence of PCR detection of viral RNA in nasal washes collected on days 5 and 10 post-contact (data not shown).

## Discussion

The differences observed between the same formulations administered either orally or intramuscularly in these animals suggest that although the oral route is expected to show a higher degree of variation among animals, this was not the case. This could be explained by the type of generated Igs. Notably, considering the i.m. administration was done in the absence of any added adjuvant, the high immunogenicity of VSP-eVLPs can be explained by the adjuvant properties of the VSP, which have been demonstrated to activate TLR-4 (19). The immunogenicity of the eVLPs lacking VSP may mainly rely on the particulate nature of eVLPs and the repetitive exposure of the antigen on their surface, even TLR-signaling as been described (41).

Our results first show that it is possible to co-express SARS-CoV-2 envelope proteins together with *Giardia* VSPs on eVLPs to generate mucosal Igs, including IgA, and NAbs against SARS-CoV-2 after oral administration. While plain eVLPs did not generate any Ab responses, VSP-decorated eVLPs (VSP-eVLPs) generated Ab responses in the range of, if not higher than, the response to i.m. administration. This may be explained by the proper conformation of the SARS-CoV-2 Env proteins at the VLP surface, which is needed for NAb production, and by the protective role of the VSP, as previously shown with influenza antigens (19). Thus, this extends our previous results with eVLP expressing HA of influenza (19), demonstrating the versatility of the VSP-eVLP platform. Actually, the dual properties of VSPs were confirmed: they not only afford protection from degradation, but also have a potent adjuvant effect. Indeed, when vaccines are administered i.m., VSP-eVLP always led to higher titers of antibodies than their plain eVLPs. We did not analyze whether VSP-specific antibodies that could be elicited after several injections of the VSP-eVLP could have a facilitating or limiting role in the induction of immune responses against SARS-CoV-2 antigens. However, VSP-eVLP boosts in VSP-eVLP previously immunized animals are effective. Of note, a SARS-CoV-2 eVLP based on our platform technology (15) was independently reported to generate a good NAb response after i.m. administration, but with no reports of IgA at mucosal sites.

Besides its ease, oral administration is known for also having the advantage of triggering better mucosal immunity. This is indeed the case here, with high levels of plasma but also bronchoalveolar lavage IgA detectable only after oral administration. This is an obvious advantage for a vaccine against SARS-CoV-2, as it should reduce viral transmission (42). It was also suggested that lack of anti-SARS-Cov-2 IgA might represent a possible cause of COVID-19 severity (43). In this line, SARS-CoV-2 was still detected in BAL of i.m. vaccinated macaques that otherwise appeared protected from infection. Whether a better mucosal response, as afforded by VSP-eVLPs, will completely sterilize challenged macaques requires further investigation.

We have not tested the specific T cell response in this study. However, it is known that eVLPs do induce robust cellular responses; indeed, using VSP-HA-eVLPs, a strong cytotoxic T lymphocyte response was generated that was able to kill HA-expressing tumor cells (19). Moreover, the IgG and IgA responses here are notoriously T cell-dependent and the good antibody response thus attests to a good T cell response (44). In this line, we previously showed that the fusion of a viral peptide to Gag, the retroviral protein precursor that drives the formation and release of the viral particle/eVLPs, produces additional strong T cell responses against this peptide (12). The fusion to Gag of large fragments or the SARS-CoV-2 N structural protein, or a stretch of immunodominant and/or conserved peptides, would be a mean to further enhance the immunogenicity of VSP-eVLPs and enhance protection against variants.

SARS-CoV-2 eVLPs and VSP-eVLPs could be used as a stand-alone vaccine, likely with a prime-boost scheme of administration. VSP-eVLPs are thermostable (19), retaining their properties at room temperature and tolerating several freeze-thaw cycles, and could thus be particularly advantageous for vaccination in countries where refrigeration of vaccine supplies is problematic. VSP-eVLPs could also be used as a boost for other vaccine designs. In this regard, it is still unknown how long the protection afforded by the currently used vaccines will last. The follow-up of infected patients indicates that, at least for some patients, the persistence of NAbs and the duration of protection might last a few months (45). These findings, plus the advent of viral variants, make it likely that the global population will need to boost the immune response of vaccinees regularly. For some vaccine designs, and particularly those based on adenoviral vectors, the re-administration of the same vector might not be very efficient due to the strong immune response generated against the vector. For these, a boost with VSP-eVLPs might be particularly interesting as they may enhance pulmonary mucosal response. For other vaccine designs, and especially if repeated administrations are needed over the years, an orally administered vaccine might be more acceptable.

The SARS-CoV-2 pandemic calls for vaccination of very large groups of people. This requires a suitable production of vaccine with an excellent safety profile. Noteworthy, we contributed to the design of an anti-CMV eVLP vaccine based on our eVLP platform that has already been used in patients, demonstrating scalable GMP production and an excellent safety profile (15, 17, 46).

Altogether, given the specific issues of each vaccine design (thermostability, side effects, lack of mucosal immunity induction, immunogenicity against the vector, among other benefits), the availability of multiple vaccines against SARS-CoV-2 improves our chances of controlling the pandemic. In this regard, a thermostable orally administered eVLP vaccine will be a valuable addition to the current arsenal against this virus.

## Data Availability Statement

The raw data supporting the conclusions of this article will be made available by the authors, without undue reservation.

## Ethics Statement

The animal study was reviewed and approved by the Institutional Committee for Care and Use of Experimental Animals.

## Author Contributions

HL and DK conceived the project. BB, HL, and DK designed the experiments, and supervised students and technicians. AS, LL, and CM performed experiments, generated plasmids, statistical analysis and figures assembly. BB, HL, and DK wrote the paper. All authors contributed to the article and approved the submitted version.

## Funding

This work was supported by grants from FONCYT (PICT-E 0234, and PICT-2116), CONICET (D4408), and UCC (80020150200144CC) of Argentina to HL, and recurrent funding by Sorbonne University and INSERM to DK.

## Conflict of Interest

HL and DK are inventors of a patent application claiming orally administered vaccines against coronaviruses that is owned by their public institutions.

The remaining authors declare that the research was conducted in the absence of any commercial or financial relationships that could be construed as a potential conflict of interest.

## Publisher’s Note

All claims expressed in this article are solely those of the authors and do not necessarily represent those of their affiliated organizations, or those of the publisher, the editors and the reviewers. Any product that may be evaluated in this article, or claim that may be made by its manufacturer, is not guaranteed or endorsed by the publisher.
